# Supplementary material for: Applications of Machine Learning in Palliative Care: A Systematic Review
Source: Cancers (Basel). 2023 Mar 4;15(5):1596. doi: 10.3390/cancers15051596 (PMC10001037; doi:10.3390/cancers15051596)
Supplement: Supplementary file 1 [file cancers-15-01596-s001.zip › Supp_Table S1.pdf]

Authors	Title	Year	Reason for Exclusion
Parikh et al.	Clinician perspectives on machine learning prognostic algorithms in the routine care of patients with cancer: a qualitative study	2022	No ML focus (meta-research)
Ruiz et al.	Early prediction of clinical deterioration using data-driven machine-learning modeling of electronic health records	2021	No human palliative care focus (cardiac surgery)
Tan et al.	Personalised, Rational, Efficacy-Driven Cancer Drug Dosing via an Artificial Intelligence SystEm (PRECISE): A Protocol for the PRECISE CURATE.AI Pilot Clinical Trial	2021	No original article (protocol)
Bao et al.	CEUS-Based Radiomics Can Show Changes in Protein Levels in Liver Metastases After Incomplete Thermal Ablation	2021	No human palliative care focus (mice)
Secunda et al.	Evaluation of automated specialty palliative care in the intensive care unit: A retrospective cohort study	2021	No ML focus
Elboga et al.	An automated synthesis of 177Lu-EDTMP as an efficient bone-seeking therapeutic radiopharmaceutical	2021	No ML focus
De Panfilis et al.	AI-based clinical decision-making systems in palliative medicine: ethical challenges	2021	No original article (review)
Rusin et al.	Automated Prediction of Cardiorespiratory Deterioration in Patients With Single Ventricle	2021	No human palliative care focus (cardiac surgery)
Bange et al.	Implementing automated prognostic models to inform palliative care: more than just the algorithm	2021	No original article (editorial)
Wegier et al.	mHOMR: a prospective observational study of an automated mortality prediction model to identify patients with unmet palliative needs	2021	No ML focus
Torenholt et al.	Between a logic of disruption and a logic of continuation: Negotiating the legitimacy of algorithms used in automated clinical decision-making	2021	No original article (editorial)
Saunders et al.	mHOMR: the acceptability of an automated mortality prediction model for timely identification of patients for palliative care	2021	No ML focus
Peruselli et al.	Artificial intelligence and palliative care: opportunities and limitations	2020	No original article (editorial)
Mehta et al.	Effectiveness of Radiofrequency Ablation in the Treatment of Painful Osseous Metastases: A Correlation Meta-Analysis with Machine Learning Cluster Identification	2020	No original article (meta-analysis)
Linnen et al.	Postimplementation Evaluation of a Machine Learning-Based Deterioration Risk Alert to Enhance Sepsis Outcome Improvements	2020	No human palliative care focus (infectiology)

Kim et al.	Artificial intelligence and lung cancer treatment decision: agreement with recommendation of multidisciplinary tumor board	2020	No human palliative care focus (oncology)
Greenwald et al.	Implementing Automated Triggers to Identify Hospitalized Patients with Possible Unmet Palliative Needs: Assessing the Impact of This Systems Approach on Clinicians	2020	No ML focus
Ibragimov et al.	Deep learning for identification of critical regions associated with toxicities after liver stereotactic body radiation therapy	2020	No human palliative care focus (radiation oncology)
Windisch et al.	Leveraging Advances in Artificial Intelligence to Improve the Quality and Timing of Palliative Care	2020	No original article (editorial)
Ashiqur et al.	Deep learning for biological age estimation	2020	No original article (review)
Thaker et al.	Automated Big Data Analytics for the Radiation Oncology Alternative Payment Model Proposal Using a Novel Health Care Software Technology	2020	No human palliative care focus (radiation oncology reimbursement)
Storick et al.	Improving palliative and end-of-life care with machine learning and routine data: a rapid review	2019	No original article (review)
Lovo et al.	Automated Stereotactic Gamma Ray Radiosurgery to the Pituitary Gland in Terminally Ill Cancer Patients with Opioid Refractory Pain	2019	No human palliative care focus (radiation oncology)
Xiao et al.	Comparison of the MAID (AI) and CAV/IE regimens with the predictive value of cyclic AMP-responsive element-binding protein 3 like protein 1 (CREB3L1) in palliative chemotherapy for advanced soft-tissue sarcoma patients	2019	No ML focus
Wegier et al.	mHOMR: a feasibility study of an automated system for identifying inpatients having an elevated risk of 1-year mortality	2019	No ML focus
Karadaghy et al.	Development and Assessment of a Machine Learning Model to Help Predict Survival Among Patients With Oral Squamous Cell Carcinoma	2019	No human palliative care focus (oncology)
Trebeschi et al.	Predicting response to cancer immunotherapy using noninvasive radiomic biomarkers	2019	No human palliative care focus (oncology)
Picker et al.	A Randomized Trial of Palliative Care Discussions Linked to an Automated Early Warning System Alert	2017	No ML focus
Meckel et al.	Development of a [177Lu]BPAMD labeling kit and an automated synthesis module for routine bone targeted endoradiotherapy	2015	No ML focus
Cheng et al.	Correlation of apolipoprotein A-I kinetics with survival and response to first-line platinum-based chemotherapy in advanced non-small cell lung cancer	2015	No ML focus

Chen et al.	Automated Assessment of Medical Students' Clinical Exposures according to AAMC Geriatric Competencies	2014	No human palliative care focus (geriatrics)
Oliver et al.	A Brazilian experience in assisted automated peritoneal dialysis: reliable option or just a palliative therapy?	2013	No ML focus
Ladjevardi et al.	Treatment with curative intent and survival in men with high-risk prostate cancer. A population-based study of 11 380 men with serum PSA level 20-100 ng/mL	2013	No ML focus
Cross et al.	Single-ventricle palliation for high-risk neonates: examining the feasibility of an automated home monitoring system after stage I palliation	2012	No ML focus
Venzin et al.	Symptomatic treatment of ascites with a peritoneo-vesical automated fluid shunt system in a dog	2012	No human palliative care focus (dog)
Giacomotto et al.	Caenorhabditis elegans as a chemical screening tool for the study of neuromuscular disorders. Manual and semi-automated methods	2012	No ML focus
Thompson et al.	Review article: the new concept of interventional heart failure therapy--part 1: electrical therapy, treatment of CAD, fluid removal, and ventricular support	2010	No original article (review)
Musshoff et al.	An automated and fully validated LC-MS/MS procedure for the simultaneous determination of 11 opioids used in palliative care, with 5 of their metabolites	2006	No ML focus
Whittington et al.	Application of a statistics-based expert system to provide automated second opinions prior to elective surgery	1993	No ML focus
Bateman et al.	Palliation of cancer in human patients by maintenance therapy with NN'N"-triethylene thiophosphoramide and N-(3-oxapentamethylene)-N'N"-diethylene phosphoramide	1958	No ML focus
